# Patient and primary care provider experience using a family health history collection, risk stratification, and clinical decision support tool: a type 2 hybrid controlled implementation-effectiveness trial

**DOI:** 10.1186/1471-2296-14-111

**Published:** 2013-08-06

**Authors:** R Ryanne Wu, Lori A Orlando, Tiffany L Himmel, Adam H Buchanan, Karen P Powell, Elizabeth R Hauser, Astrid B Agbaje, Vincent C Henrich, Geoffrey S Ginsburg

**Affiliations:** 1 Health Services Research and Development, VA Health System, Durham, NC, USA; 2 Duke Center for Personalized Medicine, Duke University, Durham, NC, USA; 3 Institute of Genome Science & Policy, Duke University, Durham, NC, USA; 4 Duke Department of Internal Medicine, Duke University Health System, Durham, NC, USA; 5 Duke Cancer Institute, Duke University Health System, Durham, NC, USA; 6 Center for Biotechnology, Genomics and Health Research, UNC-Greensboro, Greensboro, NC, USA; 7 Center for Human Genetics, Duke University, Durham, NC, USA; 8 Durham Epidemiologic Research and Information Center, VA Health System, Durham, NC, USA; 9 Cone Health System, Greensboro, NC, USA

**Keywords:** Family health history, Cancer screening, Clinical decision support, Health services

## Abstract

**Background:**

Family health history (FHH) is the single strongest predictor of disease risk and yet is significantly underutilized in primary care*.* We developed a patient facing FHH collection tool, MeTree^©^, that uses risk stratification to generate clinical decision support for breast cancer, colorectal cancer, ovarian cancer, hereditary cancer syndromes, and thrombosis. Here we present data on the experience of patients and providers after integration of MeTree^©^ into 2 primary care practices.

**Methods:**

This was a Type 2 hybrid controlled implementation-effectiveness study in 3 community-based primary care clinics in Greensboro, NC. All non-adopted adult English speaking patients with upcoming routine appointments were invited. Patients were recruited from December 2009 to the present and followed for one year. Ease of integration of MeTree^©^ into clinical practice at the two intervention clinics was evaluated through patient surveys after their appointment and at 3 months post-visit, and physician surveys 3 months after tool integration.

**Results:**

Total enrollment =1,184. Average time to complete MeTree^©^ = 27 minutes. Patients found MeTree^©^: easy to use (93%), easy to understand (97%), useful (98%), raised awareness of disease risk (85%), and changed how they think about their health (86%). Of the 26% (N = 311) asking for assistance to complete the tool, age (65 sd 9.4 vs. 57 sd 11.8, p-value < 0.00) and large pedigree size (24.4 sd 9.81 vs. 22.2 sd 8.30, p-value < 0.00) were the only significant factors; 77% of those requiring assistance were over the age of 60. Providers (N = 14) found MeTree^©^: improved their practice (86%), improved their understanding of FHH (64%), made practice easier (79%), and worthy of recommending to their peers (93%).

**Conclusions:**

Our study shows that MeTree^©^ has broad acceptance and support from both patients and providers and can be implemented without disruption to workflow.

## Background

Family health history (FHH) has long been acknowledged as an important part of the medical examination [1]. In the current age of genomics, the importance of FHH is becoming ever more apparent. According to Francis Collins, “Virtually every human illness has a hereditary component” [2] and current professional guidelines for cardiovascular disease [3], diabetes [4], breast cancer [5], and colorectal cancer [6] among others strongly endorse FHH risk stratification to develop personalized prevention strategies. Despite this, collection and use of FHH for clinical decision making in primary care is underutilized.

Many barriers exist to the accurate and complete collection and application of FHH within the traditional primary care model. Patients are frequently unprepared to provide FHH, usually due to either lack of communication among family members or failure to appreciate its importance [7,8]. At the same time, physicians find it difficult to acquire and use FHH due to time constraints, lack of standardization, and difficulty synthesizing into actionable prevention strategies [9-12].

Self-collection tools have been shown to be as good or better than the current practice of FHH collection by medical providers [13,14]. These factors make a patient-oriented FHH collection and risk stratification tool a compelling approach for overcoming barriers and improving patient care. In 2004 the Genomedical Connection, a consortium of Duke University, the University of North Carolina at Greensboro, and Cone Health System, developed the Genomic Medicine Model (GMM). The central component of the GMM was the creation of MeTree^©^, a computerized FHH collection and decision support tool for integration into primary care clinics, a practice environment uniquely suited for widespread population impact [8]. This manuscript describes the experiences of the providers and patients at intervention clinics who used MeTree^©^ as part of a Department of Defense (DoD) (grant # W81XWH-05 1-0383) funded hybrid implementation-effectiveness controlled study.

## Methods

### MeTree^©^

MeTree^©^ is a patient-facing FHH computerized collection tool with embedded clinical decision support (CDS) for patients and providers on actionable prevention strategies and education support for collecting FHH. Patients collect their FHH, then enter it into MeTree^©^ along with other relevant personal history needed to run the integrated risk calculators. MeTree^©^ then risk-stratifies patients for five diseases (breast, ovarian and colorectal cancer, thrombosis, and hereditary cancer syndromes) and recommends risk-guided prevention strategies endorsed by evidence-based guidelines [15-24]. CDS is provided in the form of a pedigree and tabular FHH along with individualized reports- one for patients with general comments about their disease risk and points to discuss with their providers, and another for providers outlining personalized action-oriented evidenced-based prevention strategies along with details of the FHH triggers and resources for additional information. Details regarding MeTree^©^’s development and validation, including the evidence-based guidelines, risk calculators, programming and validity have been published [25].

We performed a controlled hybrid type 2 implementation-effectiveness clinical trial in three community-based primary care practices in Cone Health System, Greensboro, NC. Hybrid studies are an emerging research tool for combining studies with effectiveness and implementation outcomes; primary outcomes in type 2 studies are effectiveness and secondary outcomes address both effectiveness and implementation [26,27]. The details of the study design are reported in a previously published protocol paper [28]. The study was IRB approved by all 3 institutions and the DoD. Written and informed consent was obtained from all participants in the study.

### Setting, participants, and intervention

MeTree^©^ was integrated into the work flow of two community-based primary care clinics, while the 3^rd^ served as a concurrent control for comparison of contemporaneous screening and referral rates. Clinic practices were compensated with a small sum of money for their participation. Integration included educating patients about the importance of FHH and how to collect it, providing a FHH worksheet to facilitate collection, completing MeTree^©^ at a dedicated clinic kiosk prior to their appointment, and generating CDS output. Patient reports were given to patients immediately and provider reports were integrated into the medical record for use at the patient visit. It was left up to the patient and the provider to choose whether to act, or not, on MeTree^©^’s recommendations. A study coordinator was available for assistance.

**Figure 1:**
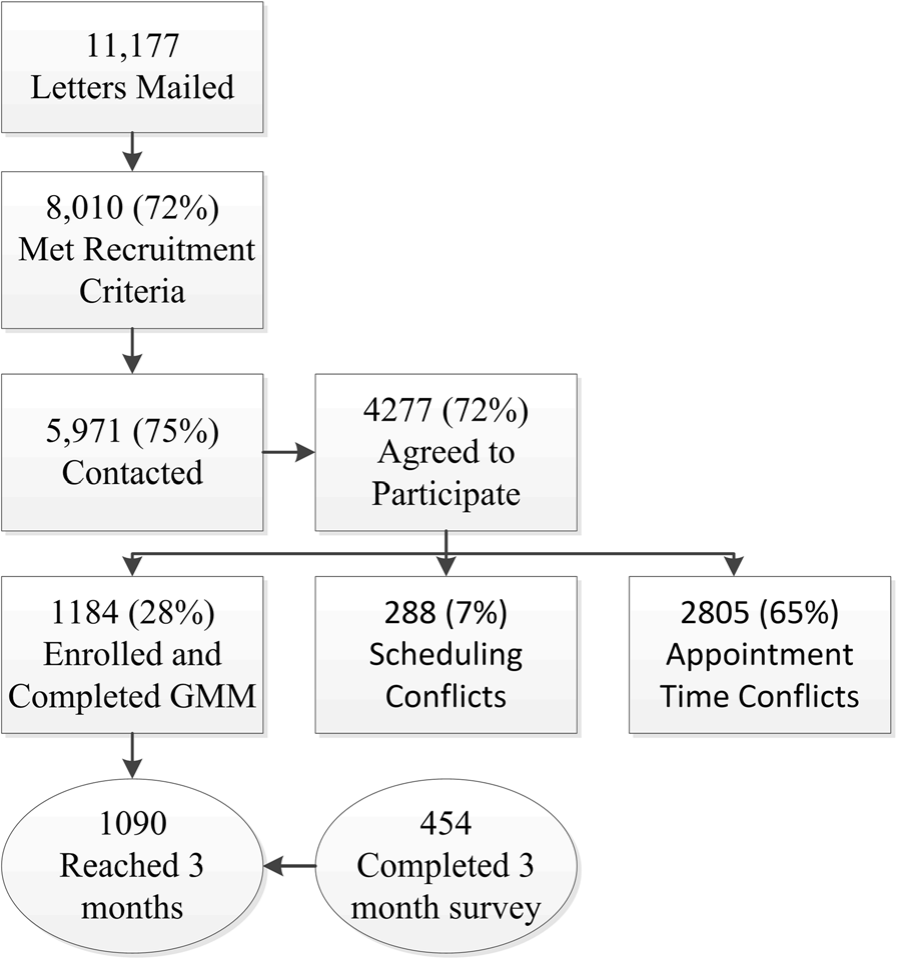
**Study flow diagram.** Previously published: Orlando LA, et al. [28].

Invitation letters were mailed to 11,177 patients with upcoming primary care well visits at the two intervention practices between October 15^th^ 2009 and April 14^th^ 2012. Children, adoptees, and non-English speakers were excluded. Patients were required to enter their FHH at the kiosk in the clinic and since only one kiosk was available at each clinic, only one person per hour time slot could enroll. See study flow diagram (Figure 1); of the 5,971 patients that were contacted by phone about the study, 4,277 (72%) agreed to participate. Of the 5,971 contacted, 1,694 (28%) declined to participate, 288 (5%) were unable to come one hour prior to their PCP appointment to complete MeTree^©^, 2,805 (47%) could not be scheduled due to only one clinic kiosk being available per one hour time slot, and 1,184 patients (20%) completed MeTree^©^.

### Outcomes and follow-up

All intervention clinic patients completed a baseline survey derived from the Health Information National Trends Survey [29] at the time of study enrollment. They were surveyed on disease risk perceptions, lifestyle, and their knowledge of health, cancer and genetics. An exit survey, completed after their primary care appointment, assessed their experience with MeTree^©^ and their discussions with their provider. At 3 months post- MeTree^©^, patients re-took the baseline survey and answered questions about MeTree^©^’s impact on their health perceptions, cancer screening practices, and discussions with family members. Physicians were interviewed prior to implementation regarding perceived barriers to integration and surveyed at 3 months post-integration regarding their experience with MeTree^©^. All surveys were paper-based and self-administered. Data were entered into RedCap by study personnel [30].

### Statistical analysis

Data was analyzed using R statistical software and all hypotheses tests were assessed at a significance level of p < 0.05 [31]. Since the intervention was allocated at the level of the clinic, to assess for the presence of data clustering, we calculated the design effect. The result, 1, indicated the absence of clustering and permitted the use of standard models without adjustment [32]. We used Pearson's chi-square test to analyze the independence of relationships for two categorical variables and ANOVA F-test for one categorical and one numeric variable. Fisher's least significant difference test assessed differences in pairs of levels. Linear regression models and t-tests analyzed the relationship of two numeric variables. When appropriate, numerical outcome variables were analyzed using multivariate analysis with standard linear regression, and categorical variables with logistic linear regression. Acceptability was evaluated based on: age, gender, ethnicity, education level, family size, and percent of family with cancer. Odds ratios for continuous variables, when used, were presented in the following units: age, per one year increase; family size, per one person increase; percent of family with cancer, per one percent increase. For multivariate analyses related to patients’ experience using MeTree^©^, a covariate regarding whether patients talked with relatives prior to using MeTree^©^ was also included. There were variable amounts of missing data. For any particular analysis individuals with missing data were dropped from that analysis. For subjects that were lost to follow-up, reminders to complete the surveys were sent three times.

## Results

### Patient characteristics

Characteristics of the 1,184 enrolled patients compared to the general clinic population are presented in Table 1. They entered information on 27,406 relatives.

**Table 1:** Baseline characteristics of patients enrolled to date as compared to the general clinic population

	**Study patients # (%)**	**Baseline clinic population # (%)**
**Patients**	1184	45000
**Gender**		
Male	490 (41.4%)	56.1%
Female	694 (58.6%)	42.7%
**Ethnicity**		
White	969 (81.8%)	75.2%
Black	159 (13.5%)	15.47%
Other	56 (4.7%)	9.4%
**Age**		
Mean (SD)	58.8 (11.79)	59.3 (13.5)
<50	250 (21.11%)	NA
50-65	575 (48.56%)	NA
>65	359 (30.32%)	NA
**Education**		
HS or less	158 (13.3%)	NA
Some college	245 (20.7%)	NA
College Deg	461 (38.9%)	NA
Any Grad	320 (27.0%)	NA
**Gail score** (SD)	0.0184 (0.01)	NA
**No. of relatives** (range)	22.89 (8–71)	NA

### Patient user experience

The user experience is presented in the following areas: ease of use, time, satisfaction, and preparedness (Table 2).

**Table 2:** **Patient experience using MeTree**^**©**^

		**N (%)**
Ease of use		
	Computer was easy to use	787/841 (93.6%)
	Questions easy to understand	855/885 (96.6%)
	Words easy to see	843/867 (97.2%)
	Received assistance to complete	311/1184 (26.0%)
Time		
	I felt rushed	18/854 (2.1%)
Satisfaction		
	Was a waste of time	12/883 (1.4%)
	Made me anxious	32/883 (3.6%)
Preparedness		
	Had enough information to complete	407/881 (46.2%)
	FHH worksheet was helpful	831/859 (96.7%)

#### Ease of use

All but 56 patients reported MeTree^©^ was easy to use and they did not feel rushed. Assistance from the study coordinator was requested by 311/1173 (26.3%) patients of whom 77% were aged over 60. Using a logistic regression model those with larger pedigrees (OR = 1.05, CI 1.03-1.07) and older age (OR = 1.07 CI 1.06-1.09) were more likely to request help.

#### Time

The average time to complete MeTree^©^ was 27.1 minutes (range 8–118, SD 12.2) with only 31/885 (3.5%) feeling they did not have enough time to complete their pedigree. On a linear regression model, patients took longer to complete MeTree^©^ when: they had talked to their relatives (coefficient 2.44, CI 1.22-3.66, p-value < 0.00), had more relatives (coefficient 0.6, CI 0.53-0.68, p-value < 0.00), or had more cancer in their family (coefficient 0.18, CI 0.12-0.24, p-value < 0.00). For example, for every increase in family size by one person, subjects took an additional 0.6 minutes (36 seconds) to complete MeTree^©^. Those who received assistance from the coordinator took less time to complete their pedigrees (coefficient −5.58, CI (−4.16)-(−7.00), p-value < 0.00). There was no difference in completion time by age, ethnicity, education, or gender.

#### Satisfaction

The majority of patients were very satisfied with MeTree^©^. Only 12/883 (1.0%) felt it was a waste of time and in multivariate analyses only “Other” ethnicity and age were significant (OR = 0.14 CI 0.03-0.71 and OR = 0.95 CI 0.90-0.99, respectively).

#### Preparedness

Most (N = 831/859, 96.7%) felt that the FHH worksheet (to guide FHH collection) was helpful; no covariates were significant in multivariate analyses. In addition, 53.9% (N = 638/1184) talked with their relatives about FHH. Patients reported that by talking to family they learned: some relatives had diseases they did not know about (N = 246/638, 38.6%); more relatives had diseases than they realized (N = 126/638, 19.7%); some relatives’ diseases were more severe than they thought (N = 70/638, 11.0%); they were mistaken about diseases some relatives had (N = 117/638, 18.3%); and how old relatives were when they got a disease (N = 186/638, 29.2%). 46% (N = 407/881) felt they had enough information to complete MeTree^©^. Those who wished they had gathered more information tended to: have a larger pedigree (OR = 0.97, CI 0.95-0.99), have less cancer in their family (OR = 1.02, CI 1.01-1.04), and were less likely to have talked with a relative (OR = 1.38, CI 1.01-1.75).

### Patient-provider discussion

Of the 1184 study participants, only 370/1184 (31.2%) answered the survey question regarding discussions with their provider (Table 3). With the exception of age, these participants are reflective of the population as a whole, with no difference in gender, ethnicity, or likelihood of having received a non-routine recommendation from MeTree^©^. Those who were younger were more likely to answer this question (mean age 57.1 vs. 59.6, p-value = 0.001), though the difference in the ages is not clinically significant. Discussions related to each CDS condition are described below.

**Table 3:** Discussions at patient-provider visits

		**All participants**	**All non-routine recommendations**	**Disease-specific non-routine recommendations**
Breast Cancer∞				
	Risk of breast cancer	48 (22.8%)	29 (23.2%)	18 (32.7%)
	Mammography	125 (59.2%)	68 (54.4%)	33 (60.0%)
	Breast MRI	10 (4.7%)	8 (6.4%)	5 (9.1%)
	Tamoxifen	6 (2.8%)	5 (4%)	4 (7.3%)
Ovarian Cancer∞		19 (9.0%)	13 (10.4%)	6 (50%)
Colon cancer*				
	Risk of colon cancer	113 (30.5%)	50 (29.2%)	39 (34.8%)
	Screening for colon cancer	260 (70.3%)	118 (69.0%)	83 (74.1%)
Thrombosis*		35 (9.5%)	18 (10.5%)	10 (41.7%)
Seeing a specialist*		82 (22.2%)	62 (36.3%)	n/a
Lifestyle choices*		192 (51.9%)	83 (48.5%)	n/a

∞Total N = 211 for all participants; 125 for all non-routine recommendations; 55 for breast cancer-specific non-routine recommendations; 12 for ovarian cancer-specific non-routine recommendations.

*Total N = 370 for all participants; 171 for all non-routine recommendations; 112 for colon cancer-specific non-routine recommendations; 24 for thrombosis-specific non-routine recommendations.

#### Breast cancer

Breast cancer risk and management was discussed in 22.7% (N = 48/211) of visits; however, the proportion was higher in patients receiving a recommendation for genetic counseling (N = 15/37 (40.5%) vs. N = 33/174 (19.0%), p = 0.004). Routine mammography was discussed in 59% (N = 125/211) of visits and was more common for those of older age (OR 1.043, CI 1.02-1.07). Breast MRI was discussed with 4% of women (N = 10/211), of which 5 had a non-routine breast cancer recommendation (i.e. breast MRI, chemoprevention, or genetic counseling referral). Those with routine recommendations for breast cancer risk were less likely to have this discussion (OR = 0.33, CI 0.09-1.19) than those with non-routine recommendations. Chemoprophylaxis was discussed in 2% (N = 6/211) of visits and was more common among those with a non-routine breast cancer recommendation (N = 2/20 (10%) vs. N = 4/191 (2.1%), p = 0.02).

#### Ovarian cancer

Ovarian cancer was discussed in 19/211 (9%) visits with female patients. Discussions of ovarian cancer were less likely if patients had a routine recommendation rather than non-routine recommendations (i.e. discussion of ovarian cancer screening or genetic counseling referral) (OR = 0.07, CI 0.02-0.25).

#### Colon cancer

Colon cancer risk was discussed in 30.5% (N = 113/370) of visits, 39 (34.8%) of which were with those who had received non-routine colon cancer recommendations (i.e. start colonoscopy before age 50, start before age 50 and perform more frequently than every 10 years, or genetic counseling referral). Discussions were more frequent with males (N = 63/159 (39.6%) men vs. N = 50/211 (23.7%) women, p = 0.001) and those with a genetic counseling recommendation (N = 21 (43.8%) vs. N = 92 (28.8%), p = 0.03). General discussions regarding colon cancer screening occurred in the majority of visits (N = 260/370, 70.3%) and were statistically independent of MeTree^©^’s recommendation.

#### Thrombosis

Thrombosis risk was discussed in 35/370 (9.5%) visits. Those with routine recommendations were less likely to have this discussion (OR = 0.11, CI 0.04-0.27) than those with non-routine recommendations (i.e. thrombosis genetic testing or genetic counseling referral).

#### Seeing a specialist

Seeing a specialist was discussed in 82/370 (22.2%) visits, and was most likely in those who had any non-routine MeTree^©^ recommendation (OR = 5.82, CI 3.21-10.54).

#### Lifestyle choices

A discussion regarding lifestyle choices occurred during 192/370 (51.9%) visits, regardless of the MeTree^©^ recommendation. Older patients (OR = 0.95, CI 0.93-0.97) and women (OR = 0.55, CI 0.35-0.86) were less likely to have such a discussion.

### Impressions of MeTree© at 3 months

At 3 months (N = 454, 38.3%) follow-up, patients still had a strongly positive view of their experience, feeling that it was helpful to them and their doctor, made them more aware of their personal risk and family health risk, and changed how they think about their health (Table 4). In addition, they would “recommend MeTree^©^ to others”. In multivariate models positive impressions were not associated with demographic factors or whether patients received a non-routine recommendation from MeTree^©^. Regarding what they “wished they knew before using MeTree^©^, 55/214 (25.7%) wished they had a greater knowledge of their FHH. Only 10 had questions regarding MeTree^©^’s CDS recommendations or their provider discussion.

**Table 4:** **Patients’ perceived benefits of using MeTree**^**©**^

		**3 months**
		**N (%)**
Respondents		454
Risk awareness		
	More aware of my risk	389 (85.1%)
	More aware of family health risk	415 (89.4%)
	Changed how I think about my health	393 (85.8%)
Usefulness		
	MeTree^©^ was helpful	403 (89.6%)
	My pedigree was helpful to me	415 (91.6%)
	My pedigree was helpful to my doctor	398 (91.7%)
	I would recommend MeTree^©^ to others	421 (92.7%)

### Providers: concerns and experience

There were 14 providers at the intervention sites, 9 physicians and one nurse practitioner at one and 4 physicians at the other. Demographics are age range 29–65 and 50% female. During pre-implementation interviews, providers indicated skepticism about the potential benefit of MeTree^©^ and concern that it would impact workflow. In particular they reported that: 1) they felt they were already collecting high quality FHHs; 2) integration of MeTree^©^ would not lead to clinically important changes in their patients’ health care plans; 3) patients would redirect discussions during the appointment towards MeTree^©^ recommendations and away from the providers’ high priority topics; and 4) MeTree^©^ would negatively impact work flow either when addressing questions or implementing recommendations.

On the 3 month post-integration survey providers were overwhelmingly supportive of MeTree^©^. In particular, they felt MeTree^©^: raised awareness about the importance of FHH and risk stratification, improved the way they practiced medicine, and made their daily routine easier; and they reported actively recommending MeTree^©^ to their peers (Table 5). In addition, MeTree^©^ made them more aware of the importance of genetic counselors and genetic counseling (81% wanted to establish relationships with genetic counselors).

**Table 5:** **Provider experience with MeTree**^**© **^**integrated into practice**

**Survey question**	**N = 14 (%)**
FHH more important now	9 (64%)
Improved practice	12 (86%)
Made practice easier	11 (79%)
Affected workflow	0%
Report was helpful	13 (93%)
Tabular pedigree was helpful	11 (79%)
Disagreed with the report^*^	0%
Recommend to peers	13 (93%)

*1 provider disagreed with 1 report (out of 1,184).

## Discussion and conclusions

Using an implementation study process to integrate the GMM resulted in tremendous support from patients and providers. This was remarkable considering physicians had a number of very strong and potentially valid concerns about a negative impact on workflow and the possibility of “hijacking” patient-provider discussions. Further, physicians often felt they were already adequately capturing FHH and its implications for disease risk in their patients’ preventive care plans. Implementation of MeTree^©^ using ongoing feedback and adaptation proved that the model could be successfully adopted within primary care, even among busy real world clinical practices. In fact these practices, as opposed to many clinical trial sites, were not early adopters who were strongly motivated to see the intervention succeed; instead they were chosen by the health system’s administration based upon their size and diversity. Clinic practices were compensated for participating, but had no pre-existing interest regarding the outcomes of the study.

While other electronic FHH and CDS tools exist, to our knowledge this is the first trial exploring direct integration of a FHH tool into real world primary care practices and the first to show that known barriers in the clinic can be successfully overcome [33-39]. The finding that patients talked with their family members, acquired new knowledge about their FHH, and changed their perception of risk, awareness, and attitude towards health supports the idea that by educating patients on the importance of collecting their FHH and its impact, the model has the potential to empower patients to take more responsibility for their care and can improve the dynamic of the patient-provider relationship. A similar improvement in risk perception and awareness has also been seen in other family history studies [33]. In addition, by providing risk stratification and actionable CDS in areas that require complex calculations and decision making with which most PCPs are not comfortable [9,10], MeTree^©^, was able to provide a valuable and time saving resource.

We examined the patient experience taking important demographic factors into account, in particular recruitment and satisfaction among minorities and undereducated. There were no significant differences in patient satisfaction in these groups. In most analyses of patient experience, age showed a small but statistically significant difference in needing more time or assistance. Recruitment and satisfaction among minorities and the under-educated was the same as the underlying population, and though age was statistically significant, the effect sizes were not clinically significant. Survey results suggest that the positive patient experience could be attributable to the extensive education available at each step in the model: collecting FHH, entering FHH into MeTree^©^, and risk assessment actions. Nevertheless we can still improve aspects around collecting FHH and talking with relatives, especially since those patients who did talk with relatives were significantly more likely to feel prepared. Focusing upon expanding tools to further improve communication among family members could have a significantly positive impact on the quality of FHH provided by patients.

MeTree^©^ may improve provider discussions on decision-making, as shown by the appropriate increase in discussions for patients at higher risk, though some MeTree^©^ recommendations were not extensively discussed, particularly those related to tamoxifen, ovarian cancer, and thrombosis. Several possible explanations exist: failure of patients to recognize the survey item representing their discussions, providers not addressing the topic because they were uncomfortable with it, or providers’ assessment that the recommendation was inaccurate or inappropriate. The latter seems unlikely, since few providers disagreed with report recommendations. One caveat is that those who answered the patient-provider discussion questions on the survey were only a subset of the study population, 31%; however, they were statistically similar to the underlying population with the exception of age, which at a difference of 2 years is clinically negligible. The low response rate may have been due to the fact that there was no option to record that none of the topics were discussed, which may have been the case for some patients, and that it was the only question located on the reverse side of the survey making it easy to miss. Another limitation is that the reliability of patient self-report of discussions with their provider is unknown. Further research will be necessary to understand the disparity between recommendations and discussions during face-to-face time between patients and providers.

An important study limitation is that implementation study designs allow adaptation to promote GMM optimization for the current setting, thus there is no assurance of generalizability. Further study across a diversity of settings is necessary to better evaluate this. In addition, several aspects of the GMM are still under evaluation. In particular we are assessing the accuracy and quality of the FHH provided as described in several previous studies [8,12,13,40-43], the impact of education on FHH collection and quality, the impact of CDS on provider care plans and on patients’ primary prevention and lifestyle behaviors, and its cost-effectiveness. While study enrollment may seem low, 72% (N = 4,277) of those contacted agreed to participate. The greatest barrier was only being able to recruit one individual per clinic per one hour time slot due to kiosk access.

Further study will offer an opportunity to obtain real world outcomes data on the potential impact of MeTree^©^ implementation on provider practice and patient behavior, both in terms of utilization of screening and genetic counseling and on lifestyle behavior of patients. Our model lays the groundwork for engaging community based practices in genomic research by outlining a model through which risk assessment and follow-up counseling and medical management occurs as a basis for implementing and evaluating a health services framework.

## Abbreviations

FHH: Family health history; GMM: Genomic Medicine Model; DoD: Department of Defense; CDS: Clinical decision support.

## Competing interest

The authors declare that they have no competing interests.

## Authors’ contributions

RRW participated in interpretation of data, drafted and critically revised the manuscript. LAO contributed to study design, interpretation of data, and critically revised the manuscript. TLH analyzed and assisted in interpretation of the data and assisted in drafting the manuscript. AHB contributed to concept and design of the study and critically revised the manuscript. KPP assisted in data acquisition and interpretation and critically revised the manuscript. ERH contributed to analysis and interpretation of the data and critically revised the manuscript. ABA contributed to conception and design of the study, assisted in data acquisition and critically revised the manuscript. VCH contributed to conception and design of the study and critically revised the manuscript. GSG contributed to conception and design of the study and critically revised the manuscript. All authors read and approved the final manuscript.

## Pre-publication history

The pre-publication history for this paper can be accessed here:

http://www.biomedcentral.com/1471-2296/14/111/prepub
